# Climate oscillations reflected within the microbiome of Arabian Sea sediments

**DOI:** 10.1038/s41598-017-05590-9

**Published:** 2017-07-20

**Authors:** William D. Orsi, Marco J. L. Coolen, Cornelia Wuchter, Lijun He, Kuldeep D. More, Xabier Irigoien, Guillem Chust, Carl Johnson, Jordon D. Hemingway, Mitchell Lee, Valier Galy, Liviu Giosan

**Affiliations:** 1 Marine Chemistry and Geochemistry, Woods Hole Oceanographic Institution, Woods Hole, USA; 2 State Key Laboratory of Estuarine and Coastal Research, East China Normal University, Shanghai, 200062 China; 3 AZTI – Marine Research, Herrera Kaia, Portualdea z/g, 20110 Pasaia (Gipuzkoa), Spain; 4 IKERBASQUE, Basque Foundation for Science, Bilbao, Spain; 5 AZTI-Tecnalia, Marine Research Division, Txatxarramendi ugartea z/g, 48395 Sukarrieta (Bizkaia), Spain; 6 Massachusetts Institute of Technology-Woods Hole Oceanographic Institution Joint Program in Oceanography/Applied Ocean Science and Engineering, Woods Hole, USA; 7 Geology and Geophysics, Woods Hole Oceanographic Institution, Woods Hole, USA; 8 Department of Earth and Environmental Science, Geobiology and Paleontology, GeoBio CenterLMU, Ludwig-Maximilians-Universität München, 80333 Munich, Germany; 9 Western Australia Organic and Isotope Geochemistry Centre (WA-OIGC), Department of Chemistry, Curtin University, Bentley, WA 6102 Australia; 10 Department of Earth and Planetary Sciences, Harvard University, Cambridge, MA 02138 USA

## Abstract

Selection of microorganisms in marine sediment is shaped by energy-yielding electron acceptors for respiration that are depleted in vertical succession. However, some taxa have been reported to reflect past depositional conditions suggesting they have experienced weak selection after burial. In sediments underlying the Arabian Sea oxygen minimum zone (OMZ), we performed the first metagenomic profiling of sedimentary DNA at centennial-scale resolution in the context of a multi-proxy paleoclimate reconstruction. While vertical distributions of sulfate reducing bacteria and methanogens indicate energy-based selection typical of anoxic marine sediments, 5–15% of taxa per sample exhibit depth-independent stratigraphies indicative of paleoenvironmental selection over relatively short geological timescales. Despite being vertically separated, indicator taxa deposited under OMZ conditions were more similar to one another than those deposited in bioturbated intervals under intervening higher oxygen. The genomic potential for denitrification also correlated with palaeo-OMZ proxies, independent of sediment depth and available nitrate and nitrite. However, metagenomes revealed mixed acid and Entner-Dourdoroff fermentation pathways encoded by many of the same denitrifier groups. Fermentation thus may explain the subsistence of these facultatively anaerobic microbes whose stratigraphy follows changing paleoceanographic conditions. At least for certain taxa, our analysis provides evidence of their paleoenvironmental selection over the last glacial-interglacial cycle.

## Introduction

In marine sediment, energy-yielding electron acceptors for microbial respiration are sequentially depleted based on the amount of energy they provide, which is linked to the oxidation of organic matter^1–3^. This process is a major factor shaping subseafloor microbial communities, which represent a substantial fraction of microbial biomass on Earth that can extend to great depths^4^. Over long geological timescales, and isolation from the “surface world”, selection in sedimentary microbial communities appears to take place, as deep subseafloor sediment exhibits a genetically distinct biosphere^5–9^.

In addition to the availability of electron acceptors for microbial respiration, the nature of deposited sedimentary material is also correlated with the diversity and activity of these communities^10^. For example, diatom-rich^11^ and sapropel^12^ layers in Pacific Ocean and Mediterranean sediments, respectively, exhibited increased rates of cellular activity. Furthermore, clay and ash layers of coastal subseafloor sediment in the Sea of Okhotsk have vastly different communities of Bacteria and Archaea^13^, and deep lignite coal layers off the coast of Japan exhibit increased rates of sulfate reduction and methanogenesis^14, 15^.

At certain sedimentary settings, some microbial taxa have been reported to be derived from taxa that were present at the time of deposition. Such groups reflect paleo-depositional conditions and this phenomena was originally referred to as “the paleome”^16^. These include continental mid-Cretaceous black shales harboring marine communities^16^, lignite-hosted subseafloor microbiomes that resemble soil communities^14^, Mediterranean turbidites harboring increased bacterial taxa from soils^17^, bacteria in Baltic Sea sediments that reflect variations in paleo-salinity^18^, and correlations between the microbial diversity and past depositional conditions in maar lake sediments^19^. These studies imply that such taxa underwent weak selection pressure after burial, either through dormancy or surviving by utilizing less-competitive substrates such as fermentation substrates (compared to higher-energy electron acceptors for respiration). Subsistence under weak selection is a hypothesis that could potentially help explain why some taxa in marine sediments correlate with environmental conditions that prevailed at the time of deposition.

In order to test this hypothesis, vertical sampling would need to be performed at relatively high resolution (*e.g*., centennial timescales) in a marine depositional setting with a history of repeated changes in paleoceanographic conditions. Such a depositional setting is found in the Arabian Sea, where continental margin sediments underlie the world’s largest oxygen minimum zone (OMZ). Arabian Sea sediment cores exhibit multiple intervals of total organic carbon (TOC) rich sapropelic intervals, whose timing coincides with millennial-scale climate oscillations recorded in Greenland ice cores^20^. δ^15^N in total organic matter, as a proxy for paleo-denitrification in the water column, was measured in multiple sediment cores from the region, and showed that the TOC-rich intervals were deposited under periods of increased OMZ strength^21, 22^. The TOC-rich intervals are separated by TOC-depleted sediments, which exhibit signs of bioturbation, and were thus deposited under higher bottom water oxygen concentrations and reduced OMZ strength^20^. Given these conditions, the Arabian Sea sedimentary record provides an excellent stratigraphic sequence of depositional settings to investigate whether some taxa in the subsurface communities exhibit a stratigraphy that reflects the past oceanographic environmental conditions.

To this end, we performed the first metagenomic profiling of sedimentary DNA at centennial-scale resolution in the context of a multi-proxy paleoclimate reconstruction. For this analysis, a 13-m long piston core spanning 52,000 years of deposition was obtained from a classical coring location within the current Arabian Sea OMZ ^20, 23–26^. Integrated analysis of these highly-resolved metagenomics and paleoceanographic datasets revealed that a subset of subsurface taxa and their metabolism correlate with the past depositional conditions over the last glacial-interglacial cycle. This supports the hypothesis that some taxa surviving with weak selection after burial can reflect their depositional environment, and thus provide novel information regarding the past oceanographic conditions. Hence, the results also provide insights into how the changing oceanographic conditions affected microbial diversity and metabolism over the last glacial-interglacial cycle in the Arabian Sea.

## Methods

### Sample collection and storage

The 13-m-long Piston core 11C spanning 52 kyr of deposition that was used for this study was obtained during *R/V* Pelagia cruise 64PE300 in 2009 from the center of the OMZ (566 m depth) on the continental slope NW of the Indus Canyon (23° N; 66° E). The core, which was originally obtained for traditional paleoclimate research, was cut in one-meter sections, capped airtight immediately, and split onboard with U-channels immediately after recovery. To prevent contamination from oxygen, core sections were sealed airtight inside the U-channels, which were then stored under anoxic conditions in the dark at +4 °C. Subsamples for geochemical and DNA analysis were obtained in 2011 under aseptic conditions inside the ancient DNA dedicated clean lab using conditions as described previously^27^. Subsamples from 12 sediment intervals were also obtained after 6 years of refrigerated storage to test whether long-term anaerobic storage at below *in situ* temperatures (10 °C)^28^ significantly affected the growth of the microbial communities. While the anaerobic storage of our samples at +4 °C was undesirable, the tests showed that there was no significant microbial growth during storage (see Supplemental Information, and Fig S1) and this is a clear indication that any resulting changes in the *in situ* microbial communities were limited. However, these tests do not control for differences in pressure experienced during sampling and storage, which may have also influenced microbial activities.

### Age model

Radiocarbon dates were obtained for mixed planktonic foraminifera or monospecific *Orbulina universa* samples (Table S1), and our age model includes calibrated radiocarbon dates for the Holocene (Table S2). Calibration was performed using Calib 7.1^29^ with a reservoir age of 565 ± 35 radiocarbon years^30^. For pre-Holocene the age model includes correlative tie points from our XRF-derived Br record in core 64PE300–11C to the high-resolution total organic carbon records of nearby core SO90-136KL^20^. Linear interpolation was used to determine ages for each individual sample.

### Bulk Geochemistry

An archived core half was scanned for bulk elemental composition using an ITRAX^TM^ micro-XRF scanner, using a molybdenum x-ray tube at an exposure time of 10 s per measurement and a step-size of 200 µm to allow for high resolution analysis of bulk chemistry^31^. The downcore variability in Br was used herein as a proxy for marine organic matter content^32^. Total organic carbon, δ^13^C, δ^15^N, and C/N were analyzed from 214 one-cm-thick sediment intervals spanning the entire record. Freeze-dried samples were first acidified to remove carbonates^33^. Samples were then transferred to an evacuated vacuum desiccator containing silica gel as a desiccant for at least 24 hours before use. A set of three standards (IAEA-N1 [Ammonium sulfate], USGS-40 [Glutamic Acid], and a well characterized glycine) plus a blank

were run between every 16 samples. NBS-19 [limestone] and a calcite laboratory standard were used to confirm bulk measurement quality. Samples were analyzed using a Carlo Erba/Fisons 1108 flash elemental analyzer (EA) equipped with a Costech “ZeroBlank” air-excluding carousel. Effluent from the EA passed via a Finnigan MAT “Conflo II” interface to a DeltaPlus stable light isotope mass spectrometer. Mass spectrometer signals (recorded using Isodat software) were used for amount determination as well as isotopic analyses. Nitrogen blanks were approximated using a method similar to that of Polissar *et al*.^33^.

### Alkenone paleothermometry

Aliquots for lipid extracts were freeze-dried and extracted using 9:1 dichloromethane/methanol (100 °C, 20 minutes), dried under ultra-high purity N_2_, and saponified using 0.5 M KOH in methanol +1% MilliQ H_2_O (15 mL, 70 °C, 1 h). Base and acid fractions were obtained by sequential liquid/liquid extraction of the saponified extract at pH = 13 and pH = 2, respectively. Alkenones were isolated from the base and acid fractions by amino-propyl-silica-gel column chromatography (eluted with Hex/DCM 4:1), combined, and further purified by silver-nitrate-silica-gel chromatography. Alkenones were anlysed by GC-FID on a Hewlett Packard 5890 equipped with a VF-1MS column (60 m × 0.25 mm I.D.). Alkenones were identified and quantified by comparison with FID traces of pure alkenone extracts obtained using the same analytical conditions. The U^k’^_37_ index was calculated following Prahl and Wakeham^34^, and sea surface temperatures (SSTs) were calculated using the global calibration of Müller *et al*.^35^.

### Ramped PyrOx measurements

For ramped oxidation, samples were loaded into a pre-combusted (850 °C, 8 hours) quartz reactor and placed in a programmable temperature oven similar to that described previously^36, 37^. An atmosphere of helium and oxygen (92% He, 8% O_2_) was passed through the reactor at 35 ± 0.5 mL/min while the sample temperature was continuously ramped at 5 ± 0.2 °C/min from 150 °C to 900 °C. Eluted gas was passed over a catalyst consisting of copper, platinum, and nickel and held at 800 °C before entering an infrared gas analyzer (IRGA, Sable Systems CA-10) for continuous CO_2_ concentration measurement. Gas was then passed into one of two cryogenic traps to isolate and quantify CO_2_ over user-defined temperature ranges. Carbon stable isotopes (δ^13^C) were measured using an Optima dual-inlet isotope ratio mass spectrometer with uncertainty below 0.1‰.

### DNA extraction

Sediment samples 2 cm thick were sampled, and a total of five grams of wet weight sediment were extracted inside the ancient DNA-dedicated lab at Woods Hole Oceanographic Institution (WHOI), aseptically as described previously^27^ and transferred into 50 mL sterile tubes. The sediments were homogenized for 40 sec at speed 6 using a Fastprep 96 homogenizer (MP Biomedicals, Santa Ana, CA) in the presence of beads and 15 ml of preheated (50 °C) sterile filtered extraction buffer (77 vol% 1M phosphate buffer pH 8, 15 vol% 200 proof ethanol, and 8 vol% of MoBio’s lysis buffer solution C1 [MoBio, Carlsbad, CA]). The extraction was repeated with 10 ml of the same extraction buffer but without C1 lysis buffer^38^. After centrifugation, the supernatants were pooled and concentrated to a volume of 100 μl without loss of DNA using 50,000 NMWL Amicon® Ultra 15 mL centrifugal filters (Millipore). Co-extracted PCR-inhibiting humic acids and other contaminants were efficiently removed from the concentrated extract using the PowerClean® Pro DNA Clean-up Kit (MoBio). The exact same procedures were performed in triplicate without the addition of sediment as a control for contamination during extraction and purification of the sedimentary DNA. Aliquots of these “extraction blanks” were run in parallel during quantitative PCR reactions.

### rRNA gene amplification, quantification, and bioinformatic analysis

DNA was quantified fluorometrically using Quant-iT PicoGreen dsDNA Reagent (Invitrogen). The V4 region of predominantly bacterial 16S rRNA genes was amplified with universal prokaryotic primers after Caporaso *et al*.^39^. The same universal reverse primer 806r (5′-GGA CTA CHV GGG TWT CTA AT-3′) was used in combination with forward primer 519f (5′-CAG CMG CCG CGG TAA-3′)^40^. Archaeal 16S rRNA genes were amplified separately using the Domain-specific primers Arch21F (5′-TTC CGG TTG ATC CYG CC-3′)^41^ and Arch915r (5′-GTG CTC CCC CGC CAA TTC-3′)^42^, and then reamplified (nested PCR) prior to sequencing using the universal primers for the V4 region after Caporaso *et al*.^39﻿﻿^. 16S rDNA (V4-region) of the widespread oceanic unicellular pelagic cyanobacterial genera *Synechococcus* and *Prochlorococcus* was amplified from the sedimentary DNA with the universal bacterial primer 806r in combination with a newly developed specific forward primer SynPro525f (5′-CGC GGT AAT ACG GGA GTG-3′). qPCR was performed using a SYBR®Green I nucleic acid stain (Invitrogen) and using a Realplex quantitative PCR system (Eppendorf, Hauppauge, NY). The annealing temperature was set to 61 °C for bacterial, 62 °C for cyanobacterial, and 63.5 °C for archaeal PCRs and reactions were stopped in the exponential phase. 16S rRNA gene libraries were sequenced on an Illumina MiSeq sequencing using the facilities of the W.M. Keck Center for Comparative and Functional Genomics, University of Illinois at Urbana-Champaign, IL, USA. The separately sequenced bacterial and archaeal libraries resulted in approximately 18 and 14 million DNA sequences, respectively. The cyanobacterial amplicons were subjected to Illumina sequencing to verify the specificity of the qPCR, which confirmed the specificity of the primers for *Prochlorococcus* and *Synechococcus*.

rRNA gene sequences were processed in QIIME^43^ (see Supplemental Methods). Reads passing quality control (removal of any sequence containing an ‘N’, minimum read length 250 bp, minimum Phred score = 20) were organized into OTUs sharing 97% sequence identity with UCLUST^44^ and assigned to taxonomic groups through BLASTn searches against the SILVA database^45^. OTU tables were rarified in QIIME using the single_rarefaction.py command to the sample with the least number of sequences (14,700 sequences), and all OTUs containing less than one sequence were removed. OTUs that were detected in only one sample were also removed. Ecological statistics were calculated in R using a Bray Curtis distance in the Vegan package (https://cran.r-project.org/web/packages/vegan/index.html). Analysis of Similarity (ANOSIM) was carried out using 999 permutations with a Bray Curtis distance. Indicator Species Analysis (ISA) was performed in the Vegan package and significance was tested with a nonparametric procedure involving the Monte-Carlo permutation procedure with 999 permutations.

### Metagenomic analysis

**Figure 1:**
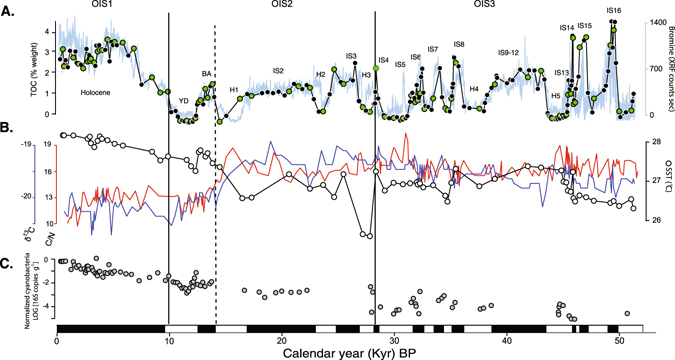
(**A**) Profiles of TOC and bromine, green circles indicate samples chosen for unamplified metagenomic sequencing. (**B**) Sea surface temperature (derived from the U^K’^_37_ index), C/N and δ^13^C profiles. Sapropelic (black) and organic-poor (white) intervals are depicted at bottom. YD: Younger Dryas, BA: Bølling-Allerød, IS: interstadial, H: Heinrich stadial, OIS: oxygen isotope stage. (**C**) Normalized qPCR quantification of cyanobacteria (predominantly *Synechococcus*) 16S rRNA gene copies. Measurements are logarithmically transformed, after converting to a percentage of total bacterial 16S rRNA genes. Note that cyanobacteria decrease exponentially (R^2^ = 0.89, P < 0.001) as a function of time. Solid lines separate oxygen isotope stages (OIS), and the dotted line marks the onset of the deglaciation.

In order to investigate the extent to which the metabolic potential of the microbial communities also correlated with past oceanographic conditions, we selected 72 of the 214 DNA samples for metagenomic sequencing (samples marked with green symbols; Fig. 1A). Because our DNA extraction protocol provided microgram quantities of extracted DNA, we could directly sequence the (unamplified) DNA. Metagenomes were directly sequenced bi-directionally on an Illumina HiSeq, at the University of Delaware Sequencing and Genotyping Center (Delaware Biotechnology Institute). Bioinformatics was performed as described in Orsi *et al*.^46^. To improve reliability of gene finding via longer alignments, over 1.5 million contigs were assembled (see Supplemental Methods for full details) averaging 1.7 kb (±0.4 SD) in length and 9.7x coverage (±2.6 SD) (Table S4). Contigs were then assigned to bacteria (96% total reads), archaea (4% total reads), and eukaryotes (<1% total reads) prior to identification of protein encoding genes and open reading frames (ORFs). Contigs with a minimum of 5x coverage were first separated into bacterial and archaeal bins via interpolated markov modeling, tetranucleotide binning, and BLAST performed in MetaWatt^47^. ORFs were detected using FragGeneScan^48^, and protein homologs were identified through BLASTp searches against the SEED database (www.theseed.org). Only hits to reference proteins with at least 60% amino acid similarity over an alignment length >50 amino acids were considered true homologs and used for downstream analysis. Assignment of ORFs to biochemical pathway classes were made based on the SEED metabolic pathway database and classification scheme. The RPKM value for ORFs was normalized against the average RPKM values of a suite of 35 universally conserved single copy genes^49, 50^, per metagenome sample.

## Results and Discussion

### Age model, geochemistry and paleoceanographic interpretation

The age model used to determine the timing of changes in depositional and environmental conditions is based on the correlation of our XRF-derived bromine (Br) record (a biophilic halogen that binds to allochthonous marine organic matter^32^) to the high-resolution TOC record of Schulz *et al*.^20^ and radiocarbon dating of foraminiferal carbonates (Tables S1, S2). The timing of increased preservation of organic matter (OM) coincides with the warm North Atlantic millennial-scale interstadial Dansgaard-Oeschger (D/O) events initially described from Greenland ice cores and North Atlantic climate archives^51^. TOC was further elevated in sediments deposited during the Bølling/Allerød (B/A) interstadial, which marks the onset of the deglacial, reaching up to 4 wt% during the warm Holocene (Fig. 1A). In contrast, organic poor, bioturbated sediments were deposited coincident with the timing of cold stadial climates originally described from the North Atlantic (e.g. Heinrich ice rafting stages and the Younger Dryas). The TOC content correlates positively with bulk δ^15^N values (r = 0.37, *P* < 0.01), a proxy for paleo-denitrification^21, 22^. Denitrifying bacteria preferentially utilize the light isotope of nitrogen leaving behind a pool of nitrate that is enriched in ^15^N during periods of increased OMZ in the water column. As this nitrogen pool is assimilated by phytoplankton it imparts an increased δ^15^N value into their biomass which is then recorded in the sediment after the OM is deposited at the seafloor^21, 22^. Thus, in agreement with earlier studies^21, 22, 52^, our results show that OMZ conditions and denitrification mainly occurred during the warm interstadials, which led to the deposition of δ^15^N-enriched and TOC-rich (>0.5%) sediments, while no or weak OMZ during cold stadials resulted in the deposition of bioturbated TOC-poor sediments. Thus, we refer to interstadial intervals with >0.5% TOC (sapropelic) as “strong OMZ” periods and stadial intervals with <0.5% TOC (bioturbated) as “weak OMZ” periods.

Br correlated with TOC across the entire core (r = 0.65 *P* < 0.01; Fig. 1A) indicating that the majority of OM deposited at the coring location was of marine origin over the last 52 kyr. The alkenone unsaturation index^35^, suggests relatively small variation in sea surface temperature between the short-term climate shifts, but reveals a sharp drop in temperature at ~29 Ka BP corresponding to Heinrich 3 (H3) stadial, which marks the base of the marine oxygen isotope stage (OIS) 2 to marine OIS3 boundary^53^. The alkenone SST further revealed a gradual warming trend over the course of the interglacial (i.e., deglacial and Holocene; Fig. 1B). The latter warming trend was accompanied with a depletion in δ^13^C values and a decrease in C/N ratios indicative of differences in origin and degree of decomposition of the sedimentary OM^54^.

### Assessing the sedimentary DNA pool

**Figure 2:**
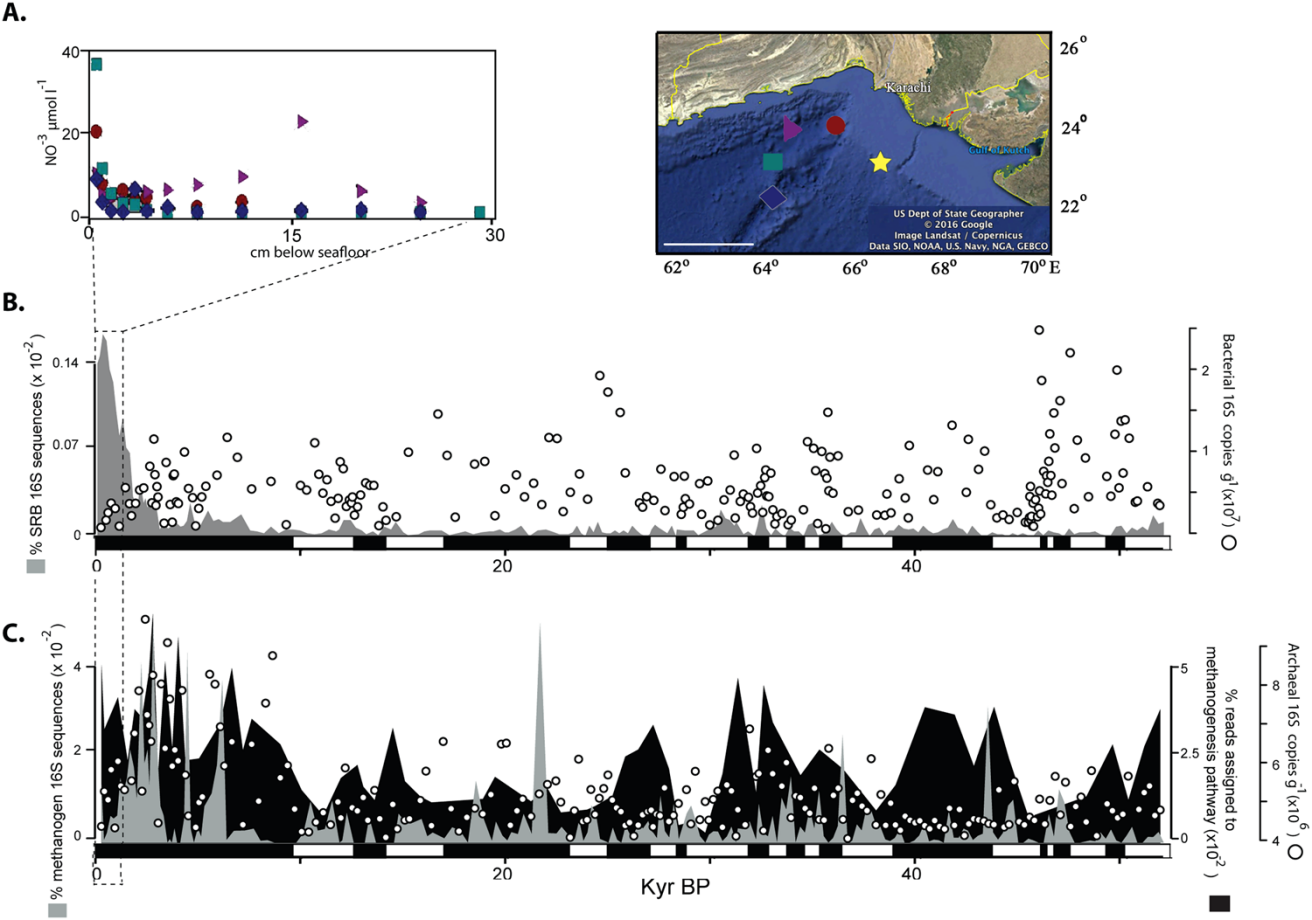
(**A**) Penetration of nitrate into the sediment surface at four nearby sites (data from Sokoll *et al*.)^62^. Star in map indicates location of the core from our study, and colored symbols indicate core locations from Sokoll *et al*.^62^. Scale bar: 200 km, map was created using the Google Earth software. (**B**) Relative abundance of 16S rRNA genes (percentage of total reads per sample) assigned to sulfate reducing bacterial (SRB) taxa across the 214 samples are shown in the grey shaded area plot, and total bacterial 16S rRNA genes as estimated by qPCR are displayed as white circles. Black and white rectangles on the x axis represent sapropelic and TOC depleted intervals, respectively. (**C**) Relative abundance of 16S rRNA genes (percentage of total reads per sample) assigned to methanogenic archaeal taxa across the 214 samples are shown in the grey shaded area plot. Relative abundances of the methanogenesis pathways in the 72 metagenomes (percentage of total reads per sample) are shown in the black shaded area plot. Total archaeal 16S rRNA genes as estimated by qPCR are shown as white circles. Note the depletion of nitrate in the top 5 cm, followed by a peak in SRB, which is directly followed by a peak in 16S rRNA genes from methanogenic archaea. Black and white rectangles on the x axis represent sapropelic and TOC depleted intervals, respectively.

Marine sediments comprise intracellular DNA associated with living and/or dead cells as well as particular bound extracellular DNA^55–58^. In order to assess to what extent prokaryotic DNA was likely to be derived from living or dead sources, we performed quantitative PCR tests. Here we targeted marine planktonic *Synechococcus*, an oxyphototrophic genus of cyanobacteria, since they require sunlight for growth and their sedimentary signal is most likely indicative of dead and decaying biomass. However, it has to be mentioned that some *Synechococcus* species, derived from freshwater, estuary, and terrestrial habitats are capable of fermentation and can subside under oxygen depleted and dark conditions^59, 60^. Our cyanobacteria qPCR primers targeted *Synechococcus* and *Prochlorococcus* (see Methods), two groups that to our knowledge have not been reported to contain marine species capable of fermentation^59^. Since our biomass is predominantly of marine source (as indicated by the comparable TOC:Br ratio) we would expect that the capability for *Synechococcus* to ferment was either not abundant or absent in our core. Indeed, despite passing through periods with different preservation conditions, *Synechococcus* 16S rRNA gene copy numbers exhibited a significant exponential decline with time (r = 0.89, *P* < 0.001), and did not correlate with higher TOC values corresponding to periods of increased OMZ strength and OM preservation (Fig. 1C). This significant exponential decline of cyanobacterial DNA with increasing sediment depth and age (Fig. 1C) shows that DNA from dead cyanobacteria had degraded as a function of time, irrespective of the preservation conditions. The half-life of the cyanobacterial DNA is approximately 4,000 years. In contrast, quantities of total bacterial and archaeal 16S rRNA gene copy numbers did not decrease significantly with sediment depth and age (*P* > 0.05, Fig. 2B,C). The lack of a decrease in the overall 16S rRNA gene copies throughout our core suggests that a substantial part of the residing prokaryotes contain intact DNA and are viable. With some notable exceptions^12, 61^, the abundance of prokaryotes in marine sediments usually decreases an order of magnitude over the top 10 m^10^. However, the multiple TOC-rich layers in our core likely bolstered the subsistence of the heterotrophic bacteria and archaea as was previously reported from Pleistocene Mediterranean sapropels^12^ and organic-rich coastal sediments^61^. Indeed, the biomass of bacteria tends to be higher in the TOC-rich layers deeper in the core (Fig. 2B).

### Vertical profiles of microbial taxonomic and functional diversity

#### Vertical distributions indicating successive depletion of anaerobic respiratory electron acceptors

Arabian Sea sediment is anoxic below the top few cm^62^. Studies from several nearby coring locations showed that nitrate is depleted within the first 10 cm^62^ (Fig. 2A). In our core, a peak in the relative abundance of 16S rRNA genes of SRB was observed between 0.2 and 0.3 mbsf (Fig. 2B). The decline in SRB at 0.3 mbsf, followed immediately by increase in methanogens (Fig. 2B,C), is suggestive of a sulfate methane transition zone (SMTZ) at ca. 0.5 mbsf. However, without measurements of dissolved sulfate and methane in pore waters we can only speculate as to the exact depth of the SMTZ. The 0.5 mbsf depth suggested by our data is within the 0.4–2 mbsf depth where sulfate reduction predominantly occurs in multiple cores from several nearby locations on the continental margin sediments of the Arabian Sea^23^. Below this depth, the metabolic potential for methanogenesis was continuously detected in metagenomes throughout the core (Fig. 2C). No genes diagnostic for sulfate reduction (dissimilatory sulfate reductase, adenosine phosphosulfate reductase) were found in the metagenomes throughout the entire core, presumably because the SRB had a relatively low (<1% total 16S rRNA gene sequences) abundance (Fig. 2B). The SMTZ depth at 0.5 mbsf is relatively shallow compared to deep drilling studies that typically report SMTZs at or below 10 mbsf^63, 64^, but similar to nearshore (<50 km offshore) coastal marine sites where the SMTZ can lie ca. 1–2 mbsf^65, 66^. While our core is further offshore (>200 km) from these nearshore sediment sites, Arabian Sea sediments exhibit some of the highest sulfate reduction rates in the world’s oceans^67^ which could explain why the SMTZ is so shallow at this site. This conclusion may seem contradictory given the relatively low abundance of SRB in the core, but per-cell sulfate reduction rates in coastal sediments can be relatively high (ca. 0.1–0.5 fmol cell^−1^ day^−1^) even when viable SRB represent 1–2% of total viable prokaryotes^66^. Below the SMTZ in marine sediments, most microbial life survives for geological timescales through fermentation of the buried organic matter^67^. Thus, below the SMTZ, which our data indicates is present in the top one meter, many of bacteria (Fig. 2B) and archaea (Fig. 2C) have likely subsisted over the last 52 Kyr via fermentation.

#### Correlation of prokaryotic communities and catabolic potential with glacial and interglacial organic matter (OM)

**Figure 3:**
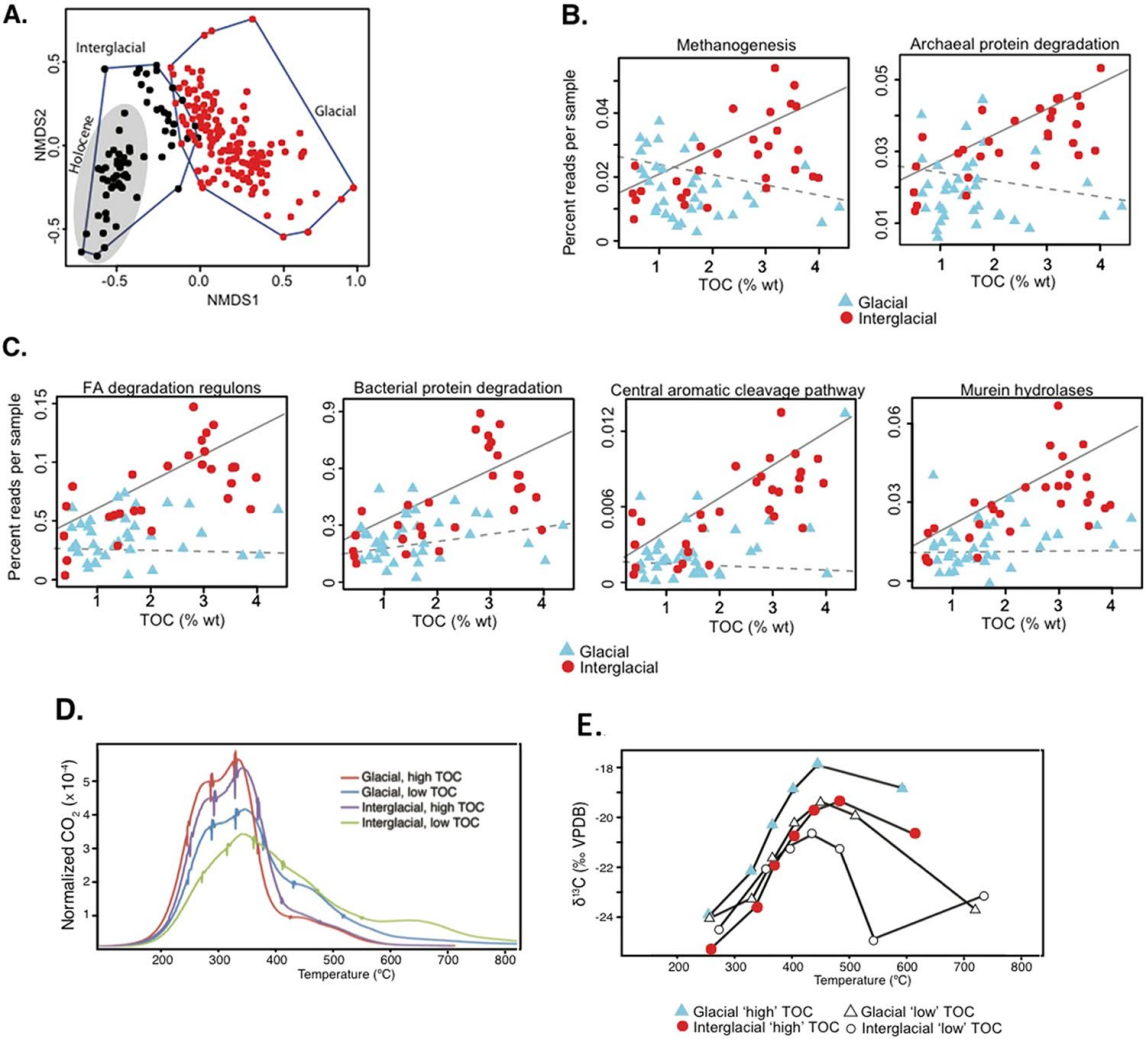
(**A**) Non-metric multidimenstional scaling analysis of 16S rRNA genes showing different microbial communities in the Holocene (grey area), interglacial (black circles), and glacial (red symbols) periods (ANOSIM: *P* = 0.001; NMDS stress: 0.12). **(B**) Correlations between SEED catabolic gene categories from Archaea and glacial v. interglacial TOC. Solid and dashed lines represent regressions for interglacial and glacial samples, respectively. (**C**) Correlations between SEED catabolic gene categories from Bacteria and glacial v. interglacial TOC. Solid and dashed lines represent regressions for interglacial and glacial samples, respectively. (**D**) Ramped PyrOx thermograms of glacial (IS4, 28 kyr) and interglacial (Holocene, 3.5 kyr) OM, and (**E**) the δ^13^C of eluted CO_2_. Thermograms are normalized such that the area under each curve is equal to unity.

The bacterial and archaeal 16S rRNA gene distribution were significantly different between glacial and interglacial sediments (Fig. 3A; ANOSIM: *P* = 0.001). While the bacterial 16S rRNA gene abundance did not correlate significantly with sediment depth, the archaeal 16S rRNA gene abundance was 5–10 fold higher in the interglacial sediments (Fig. 2C), which is mostly attributed to the Lokiarchaeota^68^, Woesearchaeota^69^, and Bathyarchaeota^70, 71^ (formerly named MBG-D, DVEG, and MCG, respectively) (Fig. S2). The relationship between OM and catabolic potential inferred from the metagenomic datasets was significantly different between glacial and interglacial intervals (Table S5). Interglacial TOC content correlated positively (Table S5) with catabolic potential for bacterial degradation of fatty acids, protein, murein, and aromatic hydrocarbons (Fig. 3C). However, this relationship was not observed for the same classes of bacterial catabolic genes when correlated against glacial TOC content (Fig. 3C). Similarly, the genomic potential for methanogenesis and archaeal protein degradation correlated positively with interglacial TOC, but not with glacial TOC (Table S5, Fig. 3B). Draft genomes of Bathyarchaeota suggest that some representative organisms are anaerobic heterotrophs and protein fermenters^70^, which is consistent with their abundance below the SMTZ (Fig. S2) that should be largely methanogenic and fermentative.

These data are consistent with the prior knowledge that the nature of deposited sedimentary material tends to correlate with the diversity and activity of microbial communities in marine sediment^10–12, 14, 15^. To investigate if the significant different gene distributions (Fig. 3A–C) in the glacial and interglacial interval is attributed to differences in lability of the OM, ramped thermal oxidation (Ramped PyrOx) of TOC from both intervals was performed. Ramped PyrOx analysis separates OM according to its thermal reactivity (a function of bonding environment) and is thus a proxy for the distribution of activation energy that characterizes OM^36^. Similar patterns of CO_2_ release (Fig. 3D) thus indicate that OM in glacial and interglacial sapropels have similar distributions of activation energy, hence similar reactivity (e.g., lability). Therefore, it is not likely that the lability of the OM is the driving factor for the significantly different gene distributions between interglacial and glacial sediments (Fig. 3A–C). Yet, the coinciding shift in the geochemical composition (δ^13^C and C/N ratio, Figs 1B and 3E, Fig. S3B) as well as a decline in haptophyte-derived alkenones (Fig. S3A) suggests that differences in the source of labile sedimentary OM (e.g. substantial species shift in the sedimentary phytoplankton biomass) might be an important trigger for the observed different microbial community composition and their catabolic capabilities between glacial and interglacial sediments.

#### Depth-independent correlations between metagenomes and the paleo-environment

**Figure 4:**
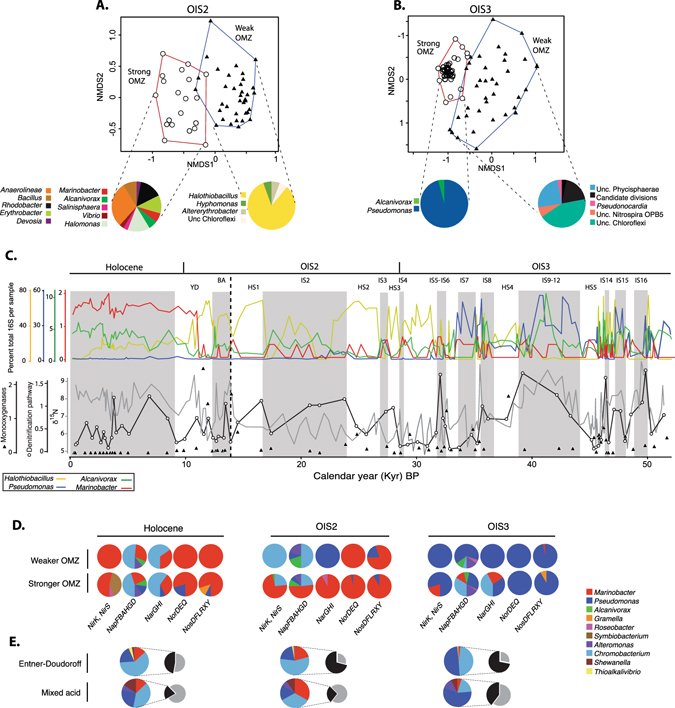
(**A,B**) Non-metric multidimenstional scaling analysis of bacterial 16S rRNA genes identified as indicator taxa through ISA analysis in OIS2 and OIS3. Pie charts show the distribution of significant indicator taxa for strong and weak OMZ periods, pie slices are proportional to the number of OTUs assigned as indicator taxa. NMDS stress values for OIS2 and OIS3 were 0.14 and 0,17, respectively. Note that despite being separated by depth, indicator taxa in sapropelic intervals (white circles) are significantly (*P* = 0.001) different compared to those in bioturbated, organic poor intervals (black triangles). (**C**) Top panel: 16S rRNA gene stratigraphy of *Halothiobacillus*, *Marinobacter*, *Alcanivorax, Pseudomonas* taxa (relative abundance). Scale shows percent sequences assigned to each taxon per sample. Bottom panel: stratigraphy of the single copy gene normalized abundance of the denitrification pathway (averaged values for *Nir*, *Nap*, *Nar*, *Nor*, *Nos*) in unamplified metagenomes (white dots), together with δ^15^N (grey line). Triangles display the average normalized abundance of monooxygenase gene homologs. The scales for monooxygenases and denitrification show the average abundance of the genes (either denitrification pathway, or monooxygenases) normalized against the averaged abundance of universally conserved single copy genes^49^. Shaded regions indicate sapropelic layers (strong OMZ) with increased TOC preservation, whereas un-shaded regions indicate bioturbated intervals deposited under higher bottom water oxygen concentrations (see Fig. 1A).  The vertical dashed line indicates the onset of the deglacial period. (**D**) Taxonomic affiliation of denitrification genes during the three major climate stages. (**E**) Taxonomic affiliation of genes encoding the mixed acid and Entner-Dourdoroff fermentation pathways (colored pie charts). Pie charts to the right show the relative abundance of Gammaproteobacterial genera encoding the two fermentation pathways (black color), and all other bacterial genera encoding the same pathways (grey color).

We performed indicator species analysis (ISA) to identify whether taxa correlated with the multiple strong and weak OMZ intervals, independent of sediment depth. ISA for the OIS2 interval identified a total of 45 (5.8%) and 69 (8.9%) OTUs as indicators for strong and weak OMZ conditions, respectively (out of 767 OTUs total). In comparison, 30 (2.2%) and 209 (15.1%) OTUs were indicators for intervals that had experienced strong and weak OMZ conditions in OIS3 (out of 1,377 OTUs total). Despite being vertically separated by several millennia, the indicator taxa deposited under recurring strong OMZ conditions are significantly more similar (ANOSIM: *P* < 0.001) to one another than those deposited under weak OMZ conditions (Fig. 4A,B, Table S3). Variable preservation of detrital DNA in sapropelic (strong OMZ) and non-sapropelic (weak OMZ) layers was not observed (Fig. 1C), and does not explain these differences.

The genera *Marinobacter* and *Alcanivorax* were relatively abundant indicator taxa for strong OMZ periods during OIS2, representing 2–5% of total 16S rRNA gene sequences per sample (Fig. 4C). The relative abundance of *Marinobacter* genes was highest during the Holocene (ca. 1.5% of total 16S rRNA gene sequences), and was repeatedly detected at similar percentages (from ca. 0.5–0.75%) throughout the core. The lack of an exponential decline with sediment depth suggests that *Marinobacter* genes did not decay as “dead DNA” over time as observed for cyanobacterial DNA (Fig. 1C). Deeper in the core *Pseudomonas* was the major indicator taxon (0–55% total 16S rRNA gene reads per sample) for strong OMZ periods together with *Alcanivorax* (0–11% total 16S rRNA gene reads per sample) (Fig. 4B), and persistently dominated in sapropelic layers of OIS3 and decreased in bioturbated intervals deposited under low OMZ strength (Fig. 4C).

Genes from the members of the strictly aerobic thiosulfate-oxidizing genus *Halothiobacillus* ^72^ were the major indicator species for periods of weak OMZ strength during marine OIS2 (Fig. 4A). *Halothiobacillus* genes representing as much as 80% of 16S rRNA gene reads in most bioturbated intervals (Fig. 4C), periods that are suggested to have had increased bottom water oxygen levels during time of deposition^52^. Their relative abundance in bioturbated intervals is greatest during OIS2 and the end of OIS3. However, deeper in the core their relative abundance in bioturbated intervals coinciding the cold Heinrich stadials 4 and 5 is markedly lower (Fig. 4C).

Metagenomes from sapropelic laminated intervals consistently exhibited a greater potential for denitrification compared to bioturbated organic depleted intervals, as evidenced by higher average relative abundance of genes in the denitrification pathway (Fig. 4C). These included the dissimilatory nitrite reductases *NirK* and *NirS*, membrane bound (*NarGHI*) and periplasmic (*NapFBAHGD*) nitrate reductases, as well as genes involved in nitric-oxide (*NorDEQ*) and nitrous-oxide (*NosDFLRXY*) respiration^73^. Geochemical studies on bulk nitrogen isotopes show that δ^15^N values above 6‰ in sedimentary OM suggest increased intensity of past water column denitrification^22^. Temporal distribution of the relative abundance of the denitrification pathway in metagenomes correlated well with δ^15^N values of bulk OM (Fig. 4C) in our core. It is reasonable to assume that the bacteria encoding the denitrification pathway in our core were more abundant during past periods of OMZ strength when δ^15^N indicates a higher intensity of denitrification. Indeed, higher abundance of denitrifier bacteria are found in the Arabian Sea OMZ compared to outside the OMZ^74^.

Metagenomic analyses revealed that the indicator taxa *Pseudomonas, Alcanivorax*, and *Marinobacter* initially detected by 16S rRNA gene profiling (Fig. 4A–C) represented the majority of denitrifying bacterial groups (Fig. 4D). The timing of the change in denitrifying community composition, from *Pseudomonas* to *Marinobacter* (Fig. 4D) coincided with the transition from OIS3 to the OIS2 full Glacial period, indicating that changing paleoceanographic conditions impacted the past succession of denitrifying communities. Compared to sapropelic layers, metagenomes from most (75%) bioturbated intervals deposited under elevated oxygenated conditions exhibited an increase in abundance of genes encoding monooxygenases (Fig. 4C). Monooxygenases are enzymes involved in cellular metabolism that require oxygen. Monooxygenases are usually encoded by aerobic and facultatively anaerobic microbes, with a notable exception being the methane monooxygenase found in the anaerobic denitrifier ‘Candidatus *Methylomirabilis oxyfera*’^75^. But, no OTUs related to ‘Candidatus *Methylomirabilis oxyfera*’ were detected in our 16S rRNA gene dataset indicating it did not contribute to the monooxygenases detected in the metagenomes. Monooxygenases were represented by kynurenine 3-monooxygenase, lysine 2-monooxygenase, lactate 2-monooxygenase, particulate methane monooxygenase, choline monooxygenase, heme-degrading monooxygenase IsdG, L-lysine 6-monooxygenase, L-ornithine 5-monooxygenase, 4-hydroxyphenylacetate 3-monooxygenase, 2,4-dichlorophenol 6-monooxygenase, tryptophan 2-monooxygenase, and alkanesulfonate monooxygenase. In contrast, in most sapropel samples monooxygenases were below detection. In line with this, the relative abundance of monooxygenases had a significant inverse correlation with preserved marine organic matter (Bromine) (r = −0.35, P < 0.001), and was thus most abundant during reduced OMZ strength.

Anaerobic ammonia oxidizing (anammox) bacteria that are present in Arabian Sea sediments under the OMZ^62^, were presumably also affected by historical changes in the OMZ strength. However, the relative abundance of the Brocadiales order, representing anammox bacteria^76^, decreased exponentially with depth (Fig. S4) and hydrazine oxidoreductase, a gene diagnostic for annamox^76^, was not detected in the unamplified metagenomes. It is possible that hydrazine oxidoreductase was not detected due to our sequencing depth (>2 million reads per sample). However, since Brocadiales are strict anammox organisms that require nitrate to obtain energy, the lack of nitrate below the top centimeters of sediment (Fig. 2A) should preclude their subsistence and explains their lack of subsistence below the upper part of the core (Fig. S4).

The distribution of denitrifier indicator taxa 16S rRNA genes is depth independent and does not correlate with pore water nitrate. Rather, they correlate with paleoenvironmental conditions namely past OMZ intensities, which indicates that a portion (5–15%) of the bacterial community in our Arabian Sea sediments have subsisted since burial with weak selection. Nitrate is depleted at the surface of Arabian Sea sediments^62^ (Fig. 2A) and the subsisting denitrifier bacteria that we observe throughout the core might not be able to use sufficient nitrate as a terminal electron acceptor. Therefore, an alternative bioenergetic pathway must support their subsistence. A significant fraction of life found below the SMTZ in continental margin sediments survive to great depths through fermentation^67^ and representatives from the indicator denitrifier taxa are known to be fermentative^77–79^. Our metagenomic analyses showed that the mixed acid and Entner-Dourdoroff fermentation pathways are encoded by many of the same groups represented by denitrifiers such as *Marinobacter, Pseudomonas*, and*, Chromobacterium* (Fig. 4D,E), suggesting a fermentative lifestyle as a potential mechanism to explain the long-term subsistence of these taxa in the absence of nitrate. Fermentation products such as hydrogen likely also play a role in fueling the activity of sulfate reducers (Fig. 2A) in a syntrophic fashion^80^. Indeed, hydrogen may play an important role in long term survival^81^.

Fermentation might also explain the subsistence of *Halothiobacillus* (Fig. 4C), whose primary metabolism as an aerobic chemolithoautotroph^73^ requires oxygen. However, *Halothiobacillus neapolitanus* for instance, is able to survive anaerobically through the heterolactic fermentation of stored polyglucose^82, 83^. Because *Halothiobacillus neapolitanus* survives on these stored compounds it is not in competition with other bacteria and would thus be under weak selection. This or similar metabolic capabilities might have preserved the correlation between *Halothiobacillus* gene stratigraphy and periods of increased bottom water oxygen (and bioturbation) in our core (Fig. 4C).

No significant differences in archaeal community structure were found between sapropelic (strong OMZ) and non-sapropelic (weak OMZ) layers indicating that, compared to Bacteria, distributions of benthic Archaea were controlled to a lesser extent by variability in Late Pleistocene OMZ strength. However, Archaea show a strong correlation with the interglacial OM deposits (Fig. 3B, Fig. S2) that has a different OM composition relative to the glacial deposits (Fig. 3D, Fig. S3). Since some anaerobic benthic archaea subsist via protein fermentation^70^, the correlations between their metabolism (Fig. 3B), abundance and diversity (Fig. S2) with the interglacial OM is likely due to the lower C:N ratios indicating increased amount of organic nitrogen in this interval (Fig. 1B).

## Conclusion

Our study provides novel insights into how marine microbial communities respond to changing oceanographic conditions over glacial-interglacial cycles at centennial timescale resolution. Our results suggest that in the Arabian Sea the diversity and metabolism of bacterial communities, in particular denitrifying bacteria and sulfur oxidizing chemolithoautotrophs, were sensitive to rapid climate-driven changes in oceanographic conditions. Because microbial activity in the Arabian Sea OMZ removes 30–50% of the ocean’s fixed nitrogen^84–86^ and also produces a significant amount of the greenhouse gas N_2_O^87^, the rapidly recurring climate-controlled selection of denitrifier communities in the Arabian Sea reported here have likely impacted past ocean biogeochemistry and potentially served as climatic feedback mechanisms. Our data support the hypothesis^88^ that certain populations of marine microbes seen today have experienced strong selection at other points in Earth’s history due to fluctuating oxygen concentrations, such as the widespread deep water anoxia of the Proterozoic oceans^89^.

Our analyses also provide highly resolved evidence that, in addition to availability of terminal electron acceptors for respiration, paleoenvironmental conditions have the potential to explain part of stratigraphic microbial distributions in marine sediment at centennial timescale resolution. This conclusion assumes that selection occurs in benthic communities that are sourced in part from the overlying water column, an assumption that is directly supported by studies that have linked the diversity of benthic microbial ecosystems to overlying source waters^90, 91^. After burial, selection should act upon populations that grow and compete in the sediments for energy-yielding substrates^2, 3, 63^. Indeed, over long time periods this has resulted in a genetically unique “deep biosphere”^5, 6, 8^. However, our study shows that 5–15% of taxa in the Arabian Sea sediments are indicators for the past oceanographic conditions, implying they are under weaker selection. As our core is only 13 m deep, we can only speculate as to how deep this phenomenon might extend. We predict that similar correlations found in other localities will be most pronounced in coastal regions sensitive to climate change, such as the Black Sea^27^ and Baltic Sea^18^.

## Electronic supplementary material

Supplementary Info
