# Supplementary material for: Climate oscillations reflected within the microbiome of Arabian Sea sediments
Source: Sci Rep. 2017 Jul 20;7:6040. doi: 10.1038/s41598-017-05590-9 (PMC5519670; doi:10.1038/s41598-017-05590-9)
Supplement: Supplementary file 1 — Supplementary Info [file 41598_2017_5590_MOESM1_ESM.pdf]

Climate oscillations reflected within the microbiome of Arabian Sea sediments

William D. Orsi[1,2,3]*†, Marco J. L. Coolen[3,4]*†, Cornelia Wuchter[3], Lijun He[5], Kuldeep D. More[4], Xabier Irigoien[6,7], Guillem Chust[8], Carl Johnson[3], Jordon D. Hemingway[3,9,10], Mitchell Lee[3], Valier Galy[3] and Liviu Giosan[11]

Affiliations:
1. Current address: Department of Earth and Environmental Sciences, Paleontology & Geobiology, Ludwig-Maximilians-Universität München, 80333 Munich, Germany.
2. Current address: GeoBio-Center[LMU], Ludwig-Maximilians-Universität München, 80333 Munich, Germany
3. Department of Marine Chemistry and Geochemistry, Woods Hole Oceanographic Institution, Woods Hole, MA 02543, USA.
4. Current address: Western Australia Organic and Isotope Geochemistry Centre, Department of Chemistry, Curtin University, Bentley, WA 6102, Australia.
5. State Key Laboratory of Estuarine and Coastal Research, East China Normal University, Shanghai, 200062, China.
6. AZTI – Marine Research, Herrera Kaia, Portualdea z/g – 20110 Pasaia (Gipuzkoa), Spain.
7. IKERBASQUE, Basque Foundation for Science, Bilbao, Spain.
8.AZTI-Tecnalia, Marine Research Division, Txatxarramendi ugartea z/g, 48395 Sukarrieta (Bizkaia), Spain.
9. Massachusetts Institute of Technology-Woods Hole Oceanographic Institution Joint Program in Oceanography/Applied Ocean Science and Engineering
10. Current address: Department of Earth and Planetary Sciences, Harvard University, Cambridge, MA 02138, USA.
11. Department of Geology and Geophysics, Woods Hole Oceanographic Institution, Woods Hole, MA 02543, USA.

* These authors contributed equally.
† Corresponding author. Email: william.orsi@gmail.com (W.D.O); marco.coolen@curtin.edu.au (M.J.L.C).

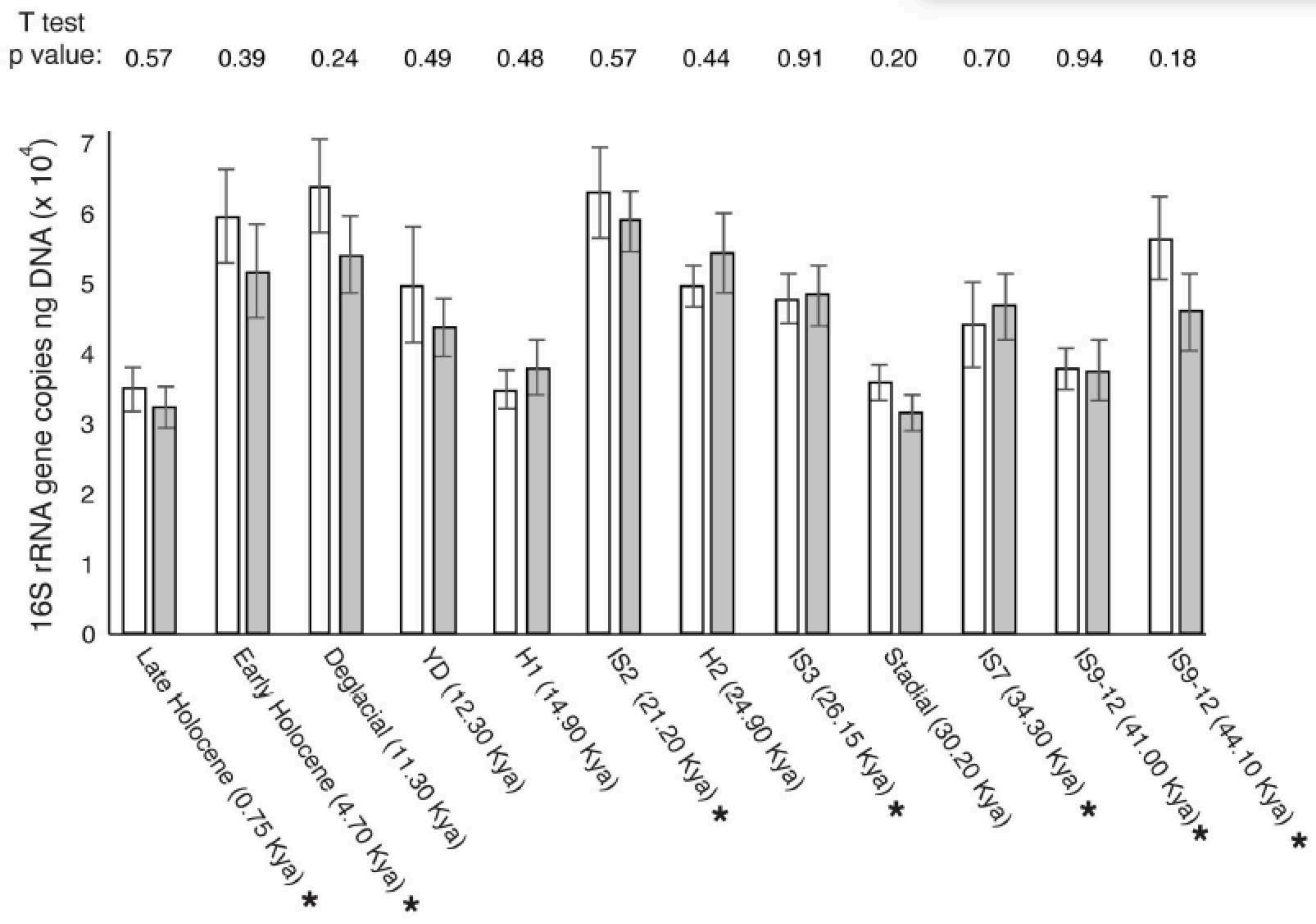

Figure S1: Estimates of microbial biomass in Arabian Sea sediment samples stored at +4 °C for 2 years (white) was compared to storage at +4 C for 6 years (grey). Histograms represent the average value across 5 replicates, and error bars represent standard deviations. If storage at +4 °C caused significant bacterial growth, it is expected that samples stored for longer periods of time at +4 °C would have significantly higher microbial biomass. However, no samples show a significant increase or decrease in the quantity of 16S rRNA genes between the different storage conditions, even in TOC rich samples. Asterisks mark sediment intervals with TOC content greater than 0.5 wt% that were deposited under suboxic conditions.

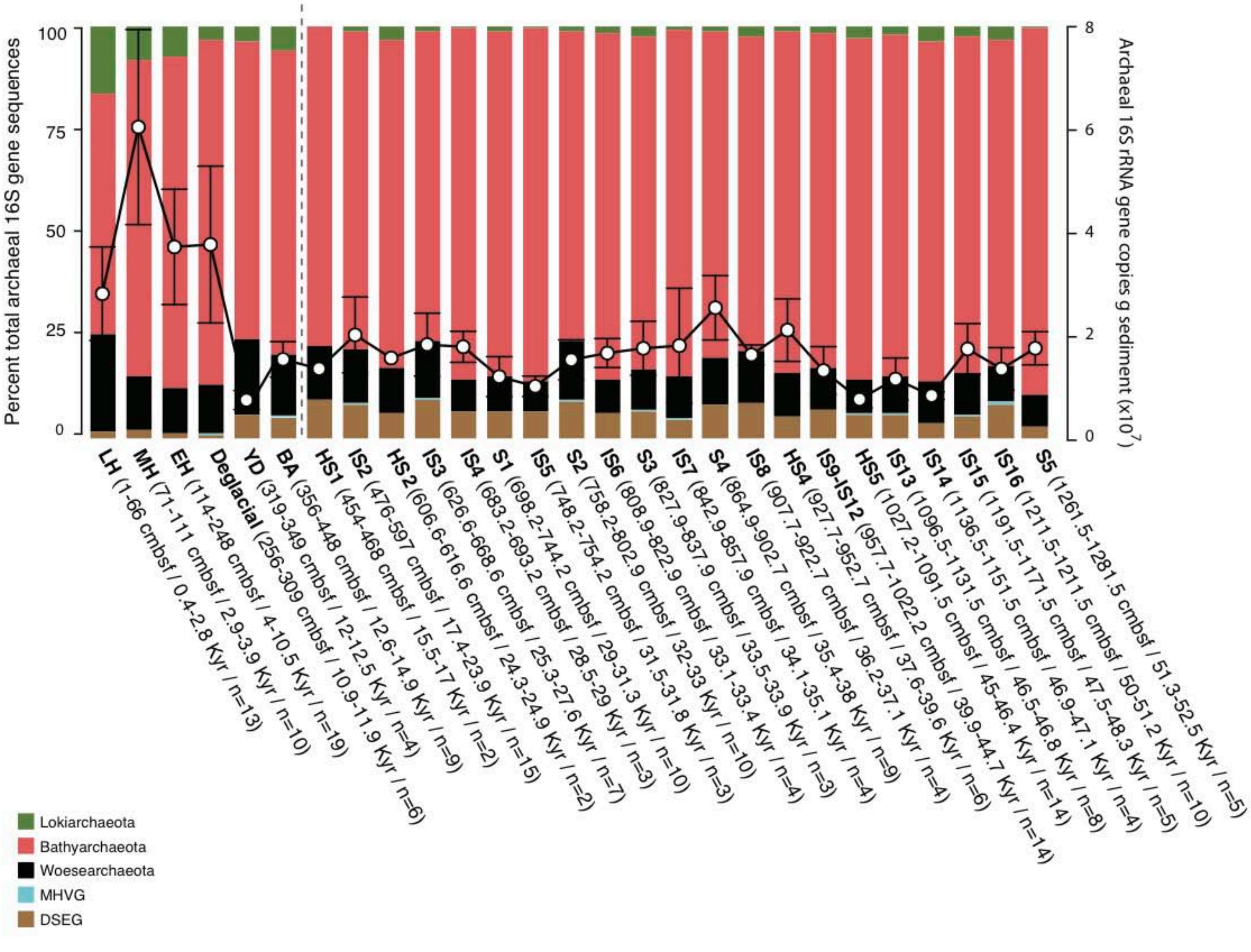

Fig S2. Taxonomic distributions of Archaea in each of the climate intervals. Climate intervals are denoted below panel B with the number of samples in each depth interval / time period / number of samples listed in brackets. The dotted line marks the Glacial Interglacial transition. Superimposed are average rRNA gene copy numbers for each climate interval. Error bars indicate the standard deviations (see bottom of Figure for number of samples in each interval, sediment depth, and time period spanned).

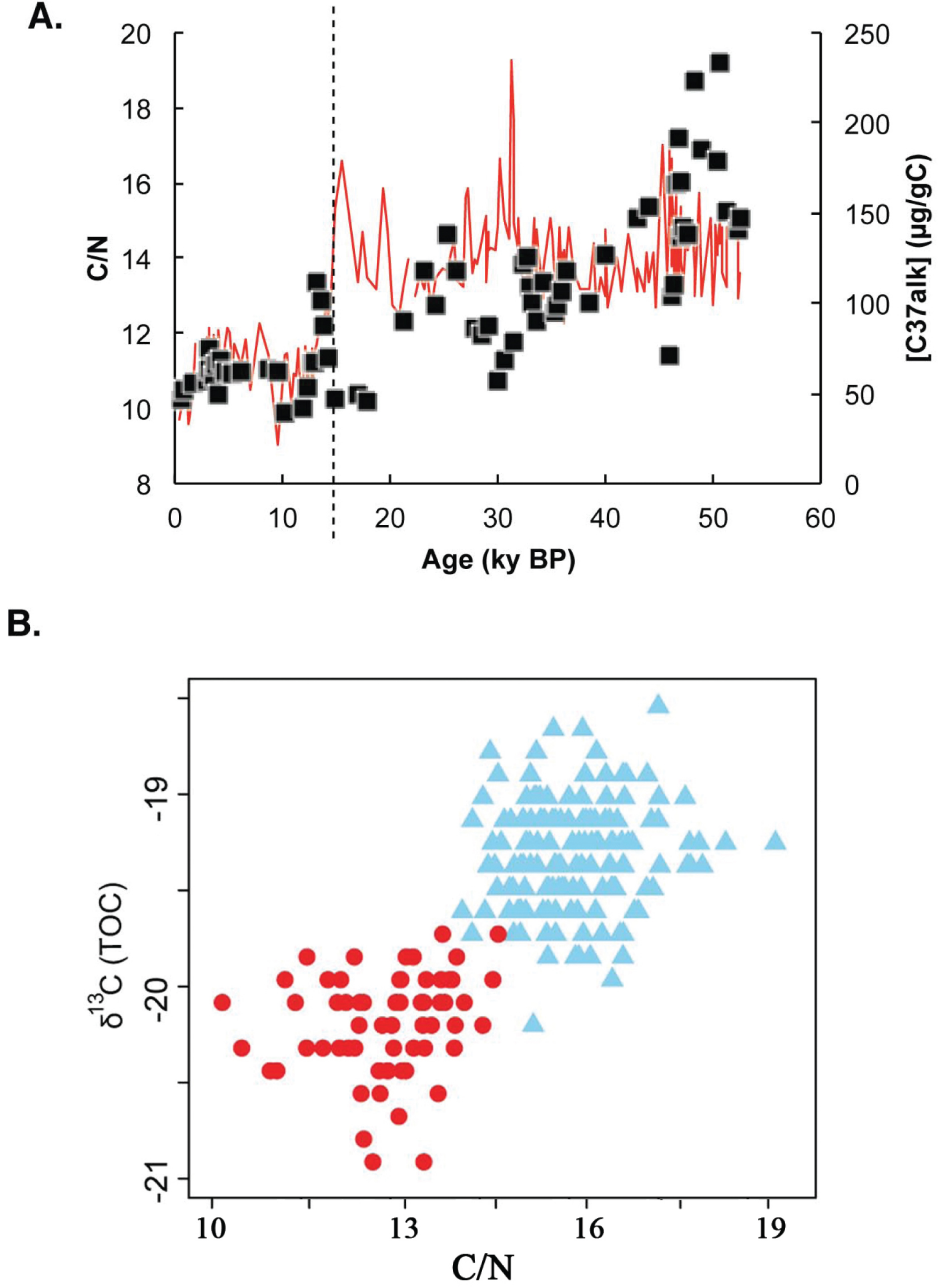

Fig. S3. (**A**) Abundance of alkenones (squares) and C/N ratios (red line). Note the relatively higher abundance of alkenones during the glacial period relative to the interglacial period. Dotted line marks the deglaciation. (**B**) OM in glacial (triangles) versus interglacial (circles) samples are chemically distinct, as evidenced by unique $\delta^{13}C$ and C/N values.

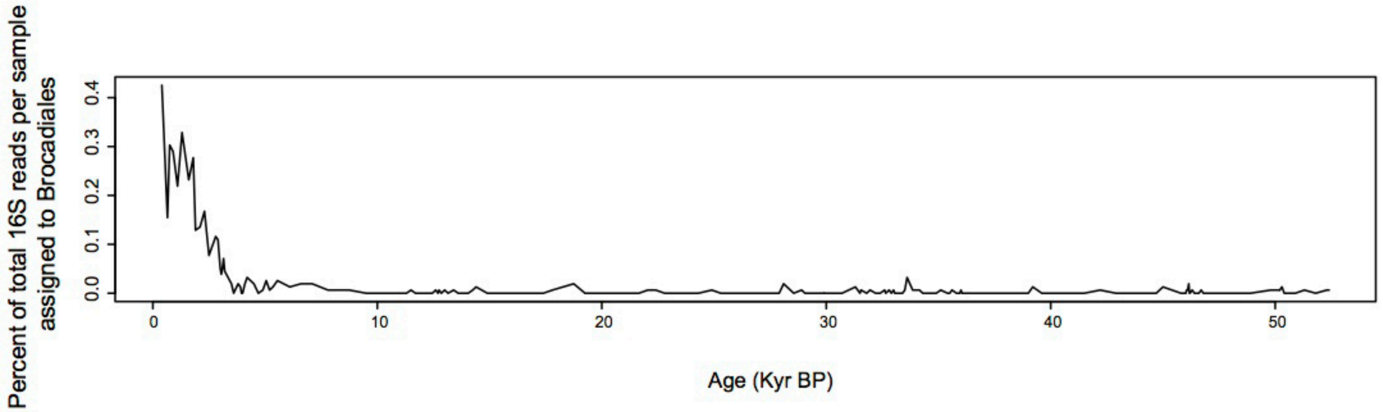

Fig. S4: Relative abundance of anammox bacteria affiliated with the Brocadiales order in the 16S rRNA gene libraries down core.

Lab No.	Depth (cm)	Species	Radiocarbon Age (^{14}C years)	Error (^{14}C years)	Calibrated Age* (cal years BP)	Median Age (cal years BP)
OS-112414	13	mixed planktonics	1470	20	745-930	848
OS-111794	73	*Orbulina universa*	3270	20	2762-2992	2877
OS-111793	87	*Orbulina universa*	3530	20	3106 - 3345	3229
OS-110013	125	*Orbulina universa*	4280	20	4043 - 4333	4172
OS-111792	176	*Orbulina universa*	5800	20	5925 - 6167	6037
OS-111795	231	*Orbulina universa*	9380	25	9879 - 10165	10030

*2 sigma error rate

Table S1: Radiocarbon dates for core 64PE300-11C.

Technique	Depth (cm)	Age cal kyr BP
Radiocarbon date	13	0.8
Radiocarbon date	73	2.9
Radiocarbon date	87	3.2
Radiocarbon date	125	4.2
Radiocarbon date	176	6.0
Radiocarbon date	231	10.0
Tie to SO90-136KL	307	12.0
Tie to SO90-136KL	374	12.8
Tie to SO90-136KL	436	14.5
Tie to SO90-136KL	453	15.4
Tie to SO90-136KL	463	16.8
Tie to SO90-136KL	599	24.0
Tie to SO90-136KL	623	25.2
Tie to SO90-136KL	674	27.8
Tie to SO90-136KL	685	28.7
Tie to SO90-136KL	703	29.2
Tie to SO90-136KL	724	30.3
Tie to SO90-136KL	760	32.1
Tie to SO90-136KL	833	33.8
Tie to SO90-136KL	869	35.5
Tie to SO90-136KL	917	36.6
Tie to SO90-136KL	935	38.5
Tie to SO90-136KL	951	39.6
Tie to SO90-136KL	970	40.4
Tie to SO90-136KL	980	41.5
Tie to SO90-136KL	995	42.8
Tie to SO90-136KL	1038	45.7
Tie to SO90-136KL	1128	46.7
Tie to SO90-136KL	1161	46.9
Tie to SO90-136KL	1177	47.6
Tie to SO90-136KL	1204	48.9
Tie to SO90-136KL	1217	50.2
Tie to SO90-136KL	1259	51.3
Tie to SO90-136KL	1276	52.4
Tie to SO90-136KL	1294	52.9

Table S2: Age model for core 64PE300-11C.

	Bacteria			Archaea		
Variable	P-value	RDA1 scores	RDA2 scores	P-value	RDA1 scores	RDA2 scores
Br	**0.01**	-0.5466	-0.5594	**0.01**	-0.6405	0.48078
%TOC	0.27	-0.4762	-0.3365	0.12	-0.4625	0.50388
%N	**0.01**	-0.5057	-0.5328	**0.01**	-0.6413	0.50305
C:N	**0.01**	-0.3143	0.6936	**0.01**	0.5972	0.53697
$\delta^{13}C$	**0.02**	0.3405	0.7366	**0.01**	0.8750	0.12247
$\delta^{15}N$	**0.01**	-0.5801	-0.1810	0.20	-0.4329	0.13044

Table S3: Redundancy analysis (RDA) for bacteria and archaea, with the significance probability value for each paleoenvironmental variable, tested using 200 permutations, and RDA scores for the first two axis. Bold font indicates significant ($P < 0.05$) correlations.

Age Kyr BP	Number of contigs	Reads mapping to contigs	Average coverage	Average length (kb)	Max contig length (kb)
0.65	18756	3388046	8.4	1.99	90
0.75	16142	2249446	7.6	1.868	105
1.60	24316	2642774	7.4	1.644	76
2.10	18451	3289249	8.5	1.923	115
2.50	19395	2613206	7.8	1.631	196
2.90	26001	3628145	8.6	1.637	122
3.10	25522	2975222	7.8	1.364	360
3.20	27306	2550554	7.7	1.274	238
3.50	26011	3127282	8.2	1.369	143
3.80	31304	4849967	9.6	1.619	223
4.00	23165	3669695	8.7	1.793	305
4.20	30922	3977837	8.5	1.583	160
4.70	21929	3908680	8.6	1.899	302
5.35	13767	4302749	10.1	2.424	628
6.10	31907	3442006	7.6	1.249	134
7.10	33570	4786439	7.6	1.534	271
8.75	28954	4541919	8.3	1.603	302
9.50	24719	3722457	8	1.416	425
10.20	28864	4993428	8.5	1.517	291
11.30	26256	5143107	8.2	1.4	491
11.90	16081	4807483	6.7	1.11	912
12.30	12224	5549052	7.1	1.575	503
12.70	21385	3836208	8.6	1.406	320
13.00	24956	7128154	8.6	1.777	444
13.15	12113	4371610	7.8	1.982	642
13.60	13327	5395632	8	1.959	1301
13.80	14218	4493735	7.7	1.701	444
14.25	18705	5147831	8.7	2.069	330
14.90	18943	4721712	7.7	1.491	445
16.95	18547	6581895	7.9	1.521	535
17.80	32698	4217451	9.7	1.568	133
21.20	21107	5363136	9.2	1.731	402
22.05	35722	6209491	9.6	1.512	187
23.10	20818	6465918	8.8	1.538	655
24.30	50724	19991574	10.3	1.2	641
25.30	9425	6518373	7.7	1.615	1250
26.15	11030	4611130	8.1	1.379	885
27.90	17753	6900444	7.5	1.389	979
28.55	7389	6305016	9.2	1.86	980
29.10	20307	4072797	9.3	1.64	329
29.90	13984	5700651	8.5	1.67	655
30.70	14691	5658677	10.1	1.64	418
31.55	36299	5343263	10	1.25	106
32.30	15583	6038014	9.2	1.8	535
32.65	34319	4218744	15	0.8	11
32.90	28142	5079237	13	0.96	504
33.10	17861	4286556	25	0.78	11
33.50	14068	5950118	13.8	1.1	68
34.30	16536	3863372	8.8	1.4	359
35.40	6681	7088076	12.7	3	628
35.60	59024	11838869	11	1.1	1160
35.95	17446	6071020	9.9	1.9	366
36.35	25024	5972118	9.6	1.7	284
38.50	21766	6164173	9.9	1.9	628
39.90	8826	5526455	15.5	2.5	402
42.90	9892	4599427	9.4	2.2	473
44.10	15213	7230125	11.2	1.8	881
45.95	28313	5230241	9.8	1.4	210
46.15	28912	5185324	12.2	1.2	155
46.40	25127	7002873	11.1	1.6	210
46.55	17783	7071887	12.4	1.9	291
46.80	12182	5020128	11.3	1.9	458
47.00	11494	7687592	9.8	1.7	642
47.05	8226	6787807	12	2.7	554
47.20	7219	6436040	7.3	1.6	1177
47.60	8763	5648347	11.6	2.8	519
48.20	21691	4754413	10	1.7	476
48.90	25046	5848677	8.4	1.3	322
50.40	16408	6073314	11.7	2.1	574
50.70	15556	5300105	10.5	2.1	673
51.20	12885	6211942	11.1	1.9	411
52.50	15588	5543548	10	1.9	350

Table S4: Assembly summary statistics for the 72 metagenomes.

Metric	Interglacial TOC		Glacial TOC	
	R-squared	P value	R-squared	P value
Methanogenesis	**0.38**	**0.0007**	0.07	0.5
Archaeal protein degradation	**0.5**	**<0.00001**	-0.01	0.46
Bacterial protein degradation	**0.38**	**0.0002**	-0.02	0.54
Bacterial FA degradation regulons	**0.42**	**<0.00001**	-0.02	0.54
Bacterial central aromatic cleavage pathway	**0.5**	**<0.00001**	-0.011	0.4
Bacterial murein hydrolases	**0.4**	**0.0001**	0.04	0.12

Table S5: Correlations of bacterial and archaeal SEED catabolic gene categories with TOC in the interglacial and glacial stages (Fig. 3B). Bold font indicates significant ($P < 0.05$) correlations.
